# Proposal for a New Differential High-Sensitivity Refractometer for the Simultaneous Measurement of Two Refractive Indices and Their Differences

**DOI:** 10.3390/s24113340

**Published:** 2024-05-23

**Authors:** Šimons Svirskis, Dmitrijs Merkulovs, Vladimirs Kozlovs

**Affiliations:** 1 Institute of Microbiology and Virology, Riga Stradins University, 16 Dzirciema Str., LV-1007 Riga, Latvia; vladimirs.kozlovs@rsu.lv; 2 ELMI Ltd., 7B Bukultu Str., LV-1005 Riga, Latvia; dmitrijs_merkulovs@yahoo.co.uk

**Keywords:** refractive index, refractometer, optical cell, environment, nano-pollution, measurement, laser

## Abstract

The refractive index of a liquid serves as a fundamental parameter reflecting its composition, thereby enabling the determination of component concentrations in various fields such as chemical research, the food industry, and environmental monitoring. Traditional methods for refractive index (RI) measurement rely on light deflection angles at interfaces between the liquid and a material with a known refractive index. In this paper, the authors present a new differential refractometer for the highly sensitive measurement of RI differences between two liquid samples. Using a configuration with two cells equipped with flat parallel plates as measuring elements, the instrument facilitates accurate analysis. Namely, the sensor signals from both the solution and the solvent cuvette are generated simultaneously with one laser pulse, reducing the possible fluctuations in the laser radiation intensity. Our evaluation shows the high sensitivity of RI measurements $<{7\times 10}^{-6}$), so this differential refractometer can be proposed not only as a high-sensitivity sensing tool that can be used for mobile detection of nanoparticles in solution samples but also to determine the level of environmental nano-pollution using water (including rain, snow) samples from various natural as well as industrial sources, thus helping to solve some important environmental problems.

## 1. Introduction

It is well known [1,2,3,4] that a differential refractometer is a specialized instrument used in analytical chemistry to measure the difference in the refractive index between two substances, typically a sample and a reference material. The refractive index determines how much the speed of light in a substance (medium) decreases compared to the speed of light in a vacuum. Differential refractometers are available for HPLC, but refractive index measurements are temperature sensitive, and good temperature control is essential when high sensitivity is required. The main advantage of the refractive index detector is its wide applicability. Under constant external conditions (e.g., temperature), the refractive index (RI) of a liquid depends on its composition; therefore, measuring the RI allows the determination of the concentrations of its components. Therefore, refractometric methods for measuring the RI are used where it is necessary to obtain information about the concentrations of solution components, that is, in chemical and physical research, in the food industry, in pollution monitoring, and in many other areas [1]. One method [2] for determining the RI n_l_ of a liquid is based on measuring the angle deflections of light rays at the interface between the liquid under study and a material with a known refractive index n_s_. The angles of incidence θ_i_ and refraction θ_r_ at the boundary are related by Snell’s law as follows:

(1) $$n_{l}\;\times \;\sin\theta_{i}=n_{s}\;\times \;\sin\theta_{r}$$

Thus, if the angle θ_r_ is measured in some way, then, with a known n_s_ and θ_i_, the value n_l_ is determined from Formula (1).

To implement the deflection method, the liquid was placed in a cuvette composed of optical glass or quartz. Cuvettes can have different geometric shapes, for example, the shape of a prismatic cavity, as in the Hilger–Chance angular deviation refractometer [5], or a cylindrical shape, as discussed in article [4,6]. Parallel collimated rays of light falling on the outer surface of the cuvette are sequentially refracted at the interfaces between the liquid and the walls of the cuvette and exit the cuvette at a certain angle relative to the initial direction. The direction of the outgoing rays and their displacement associated with the changes in the RI of the liquid are recorded using a coordinate-sensitive photodetector. Previously [7], we proposed the design of a measuring cell and a new method for measuring the deflection angle, which allowed us to determine the RI with an accuracy of ~$10^{-5}$. In [8], we applied this method to measure the concentration of polymer nanoparticles in aqueous suspensions and showed that the detection limit was ~1 mg/L.

However, to achieve this, it is necessary to maintain the temperature at which measurements are carried out with an accuracy of ~0.01 °C, which significantly complicates experiments involving the replacement of samples with different concentrations of nanoparticles. To directly determine the difference, δn, between the refractive indices of the two liquids, Brice [3] proposed a differential refractometer, which consists of a rectangular cell divided by an inclined glass plate into two prismatic compartments. This constructive idea with following basic principles was implemented in many modern refractometric devices now offered by various manufacturers like Wyatt Technologies [4], Waters [9], and Infitek [10]. The increased sensitivity of modern refractometers compared to older models is due to the use of photodiode arrays to determine the angle of deflection, which allows the sensitivity of the refractometer to be increased.

**Figure 1:**
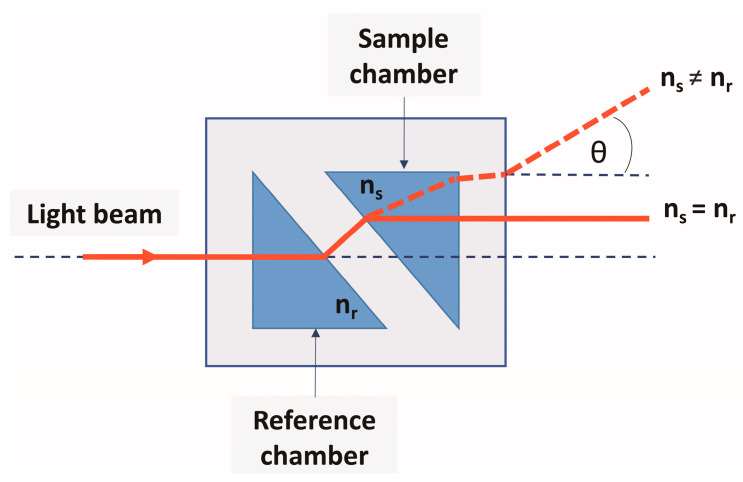
Schematic presentation of the refraction and displacement of a light beam in a differential refractometer. θ—angle of outgoing light beam; n_r_—refractive index of the reference; n_s_—refractive index of the sample.

When both compartments were filled with the same liquid, the light beam passed through the cell without angle deflection (see Figure 1). If, in one of the compartments, the RI changes by a small amount, δn, for example, owing to the dissolution of an impurity, then the beam will deviate by an angle θ proportional to δn [11]. This approach, that is, the comparison of signals when light passes through two cuvettes with liquids to determine the difference between their refractive indices, is considered in this study. The cuvettes described in [5] were used as measuring elements, and the principle and measurement scheme are presented in the next section.

## 2. Principles of the RI Measurement

Since our proposed method for measuring the refractive index is described only in conference proceedings [7,8], we consider the main details of the method below.

**Figure 2:**
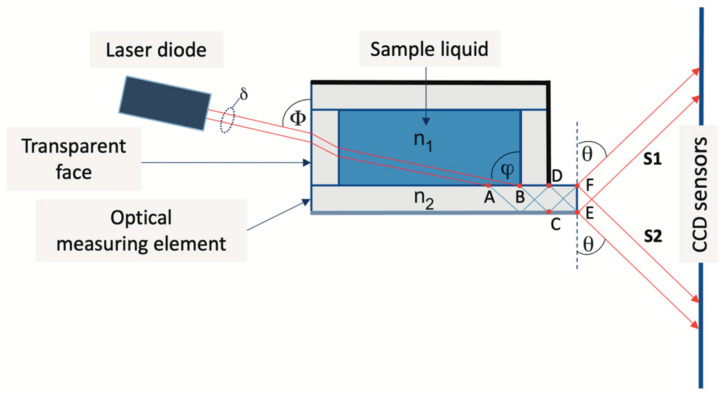
The principle of measuring the deflection angle using a plane-parallel plate as a measuring element. S1 and S2—beams of parallel light rays; δ—laser beam width; Φ—angle of incoming laser beam; φ—angle of incidence of rays on the measuring element; θ—angle of outgoing light rays; A, B, C, D, E, F—reference points; n_1_—RI of the sample liquid; n_2_—RI of the optical measuring element.

The proposed method for measuring the deflection angle [7] is illustrated in Figure 2. The optical measuring element was a rectangular plane-parallel plate with polished edges, made of optical glass with RI n_2_. The plate is part of the cuvette, for example, its wall, as in the case shown in Figure 2. A beam of parallel light rays from a laser diode is directed towards the transparent face at an angle Φ. Then, the rays pass through the cell wall, through the liquid under study with RI n_1_ and fall on the measuring optical element at a certain angle φ.

The value of angle φ is chosen such that two conditions are met. First, after refraction at the interface between the liquid and the measuring element, the total internal reflection must occur on the lower surface of the latter so that the refracted rays propagate in the measuring element as if through a waveguide, for example, with $n_{1}$ = 1.33 (water) and $n_{2}$ = 1.5, which is already satisfied at φ > 42. Since it can be shown that condition φ > Φ is always satisfied, we fix the value: Φ = 50°.

Note that the full picture of the reflected–refracted rays in the measuring cell is quite complex and is not reflected in Figure 2. In addition, an absorbing coating is applied to the two walls of the measuring cell in order to prevent scattered light from reaching the sensors. We consider only rays propagating along the measuring cell elements that form the output signals, and the total reflection is observed, naturally, only on the lower edge of the measuring element. Second, the refracted beam must be so wide that it cannot exit the plate entirely in one direction but would be divided at the exit face, EF, of the plate into two parts S1 and S2 for any $n_{1}$ < $n_{2}$. It can be shown that this occurs when the laser beam width δ satisfies the condition

δ > 2$\cdot \frac{n_{1}\cdot sin\varphi \cdot cos\varphi }{\sqrt{n_{2}^{2}-{\left(n_{1}\cdot \sin\varphi \right)}^{2}}}\cdot d$, where d is the thickness of the measuring element plate, and φ is the angle of incidence of the rays on the measuring element. Thus, for the rays that experience the last reflection from the surface of the plate in the DF section, the outgoing light beam S2 is formed, and those reflected in the CE section form the S1 beam. The angle θ, which specifies the position of the beam images on the sensor, is determined by the expression

(2) $$\theta =\pi /2-arcsin(\sqrt[{2}]{n_{2}^{2}-n_{1}^{2}+{cos}^{2}\Phi }),$$

which is obtained by considering the ray paths. Note that the value of the angle θ does not depend on the refractive index of the material of the cell walls. Expression (2) can be rewritten in the equivalent form

(3) $$n_{1}=\sqrt[{2}]{n_{2}^{2}+{cos}^{2}\Phi -{cos}^{2}\theta \;}\;.$$

The Formulas (2) and (3) were obtained based on the fact that there is an interface between the liquid under study and the measuring element, i.e., n_1_ < n_2_. Therefore, the limit for measuring the RI of a liquid is determined by the value n_2_. Within the framework of geometric optics, a stepwise abrupt increase and decrease in light intensity should be observed in the cross section of beams S1 (S2). In this case—monochromatic light and the output of rays through a narrow “slit” (face FE in Figure 2)—the signal is formed because of the interference of many rays. However, as shown below, the emerging beams have a clear boundary between light and shadows.

**Figure 3:**
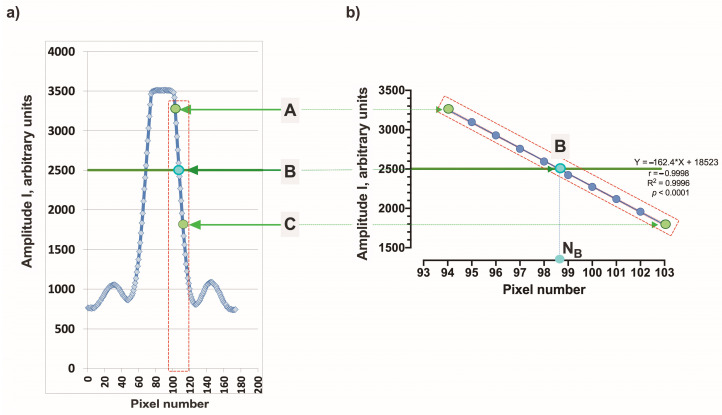
(**a**) The intensity distribution in the cross section of the light beam emerging from the measuring element; (**b**) approximation of the front of the sensor signal.

The measuring cell is designed as follows. The measuring element is a plate made of optical glass with a refractive index n_2_ = 1.518, with dimensions of 20 × 10 × 1 mm. A window of 8 × 6 mm was cut out in a standard transparent spectrophotometric cuvette made of polypropylene. The measuring element was attached to the cuvette using transparent sealant so that it covered the input window. An Edmund Optics 0.9 mW 633 nm laser diode was used as the light source. The laser operates in a pulsed mode with a pulse duration of 1 ms, and the number of pulses in a single measurement can be varied in the range (1–150) in order to select the optimal amplitude of the sensor signal. A Hamamatsu monochromatic linear 1024-pixel image sensor S9226 with a pixel width Δp = 0.0078 mm is used. In Figure 3a, the characteristic shape of the sensor signal is shown when recording the light beams emanating from the measuring element.

When the RI $n_{1}$ of the liquid changes, the angle θ (see Figure 2) changes, the image of the signal from the outgoing rays moves across the sensor, and the shape of the signal does not change [8]; that is., we can say that there is a “parallel transfer” of the signal across the sensor. Therefore, to determine the position of the signal image on the sensor, it is generally sufficient to indicate the coordinates of any selected point B, which we select as follows. Let us consider the section AC of the right edge of the signal (see Figure 3a), which includes signals from pixels whose readings lie in the interval (0.8 − 0.5) × $I_{max}$, where $I_{max}$ = 3500 is the maximum value of amplitude I of the sensor signal. This interval contains readings from 8 to 10 sensor pixels. Processing using the least squares method shows that in the AC section the amplitude I linearly depends on the pixel number N with a pair correlation coefficient > 0.999 (for example, for the signal shown in Figure 3b, I(N) = −164.2*N + 18,523). Now, we can determine the “coordinates” N_B_ of point B from the condition that the value I takes on a certain given value Ig. In the following calculations, we assumed that Ig = 2500, for which the corresponding signal coordinate N_B_ = 98.58 (Figure 3b).

## 3. Design of the Differential Refractometer and Preliminary Results

**Figure 4:**
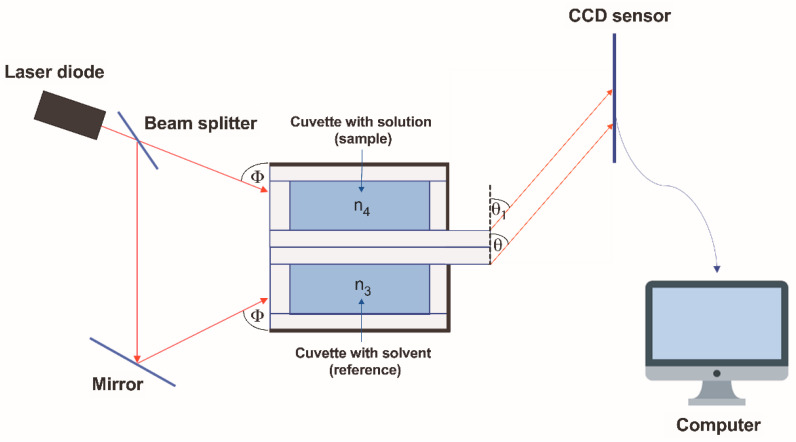
Schematic view of the proposed differential refractometer. Φ—angle of incoming laser beam; θ, θ_1_—angles of outgoing light rays; n_3_—RI of the solvent; n_4_—RI of the solution; red arrows—light rays.

The diagram of the new differential refractometer designed and proposed by our team is shown in Figure 4.

The differential refractometer consisted of two measuring cells. The first cuvette contained a solvent with a refractive index of n_3_. Another cuvette contained a solution with a refractive index of n_4_. The light beam from the laser diode was divided into two parts using a splitter, one of which was directed directly at the input window of the first cuvette. The second part is directed using a mirror to the entrance window of the second cell, and the mirror is positioned such that the angles Φ of incidence of both beams are equal to each other. The light beams emerging from the measuring elements were recorded using a CCD sensor. In this case, when n_3_ = n_4_ and θ = θ_1_, the rays emerging from the measuring elements are parallel. The distance between the sensor signals from these beams should not change when the distance between the measuring cell and the sensor changes. This fact was verified experimentally when setting up the device. All elements—laser, beam splitter, mirror, and CCD-sensor—of the refractometer are rigidly fixed; i.e., there are no moving elements in the design.

**Figure 5:**
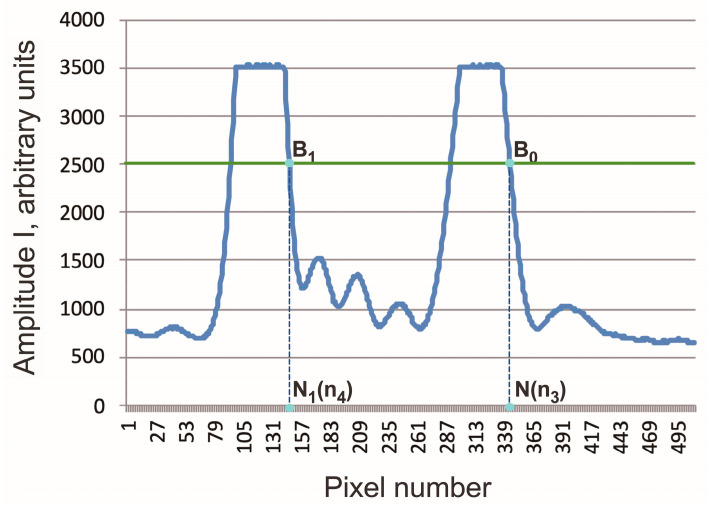
Sensor signals when recording light beams emerging from a cuvette with a solvent (right signal) and a cuvette with a solution (left signal). B_0_, B_1_—position of signals on the sensor; N(n_3_) and N_1_(n_4_)—pixel number coordinates for the position of signals B_0_ and B_1_, respectively.

The images of the signals from the sensor are shown in Figure 5. The position of the signals on the sensor is determined by points B_0_ and B_1_, the coordinates of which, N(n_3_) and N_1_(n_4_), are determined in the manner described above.

**Figure 6:**
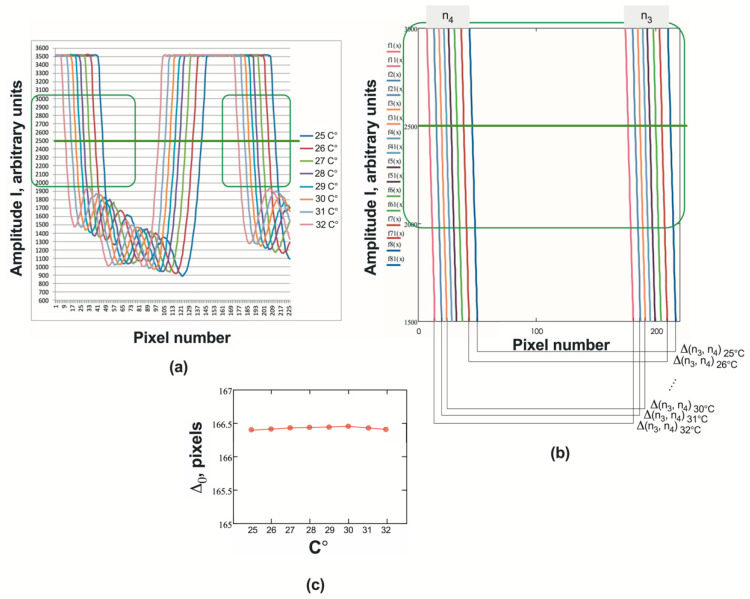
(**a**) Sensor signals at different temperatures of the sample solution; (**b**) signal edges obtained by processing using the least squares method; (**c**) values of Δ_0_ regarding temperature.

We defined the distance between the signals from the solution and the solvent as Δ(n_3_, n_4_) = N(n_3_) − N_1_(n_4_). In order to study the effect of the temperature of liquids on the measurement of Δ(n_3_, n_4_), we conducted the following experiment. Initially, both cuvettes were filled with distilled water heated to 32 °C. Then, the positions of the signals were recorded as the water under normal conditions stepwise cooled to room temperature. The results are shown in the Figure 6a,b.

As can be seen from Figure 6c, Δ(n_3_, n_4_) = Δ_0_ = const over a fairly wide range of operating temperatures: 25–32 °C. Consequently, Δ(n_3_, n_4_) changes only with a change in n_4_, that is, with a change in the refractive index of the solution. Therefore, the movement of the D signal from the cuvette with the solution, caused by a change in its composition (signal shift) can be determined by the expression: D = Δ(n_3_, n_4_) − Δ_0_.

**Figure 7:**
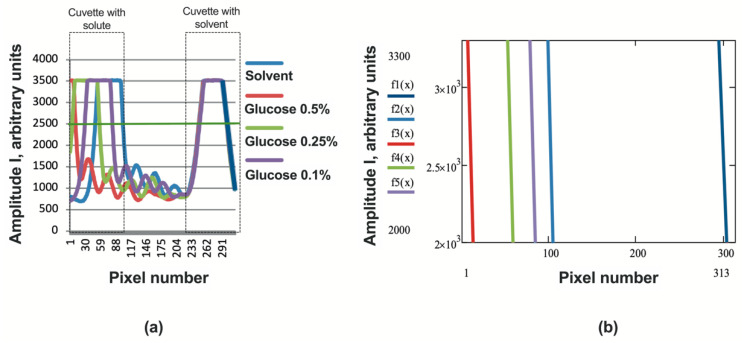
(**a**) Sensor signals at different concentrations of sugar solution; (**b**) signal edges obtained by processing using the least squares method.

**Figure 8:**
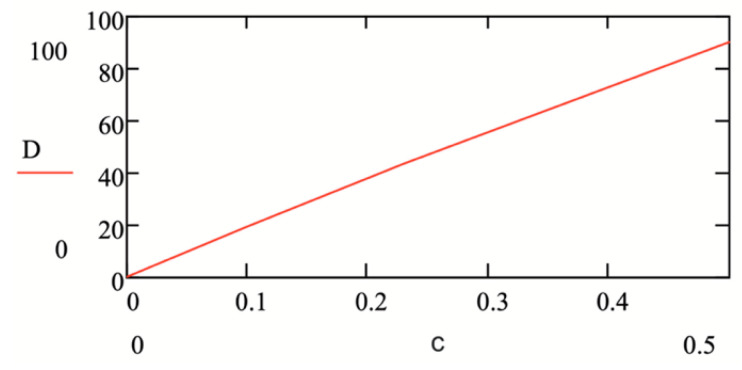
The signal shift (D, pixels) of the cell with the solution depending on the concentration (c, %) of the sugar solution.

To determine the sensitivity of the proposed refractometer, we measured the sugar solutions, the optical properties of which are well known [12]. The measurement results are shown in Figure 7a,b and Figure 8.

The increment in the RI of the 0.5% sugar solution compared to the refractive index of water was Δ$n_{4}\;$= ${7\cdot 10}^{-4}$. As can be seen from Figure 8, such a change in the RI, associated with a change in the concentration of sugar in the solution, corresponds to a shift in the signal D by 90 pixels. If we assume that the minimum recorded signal movement is one pixel, then the sensitivity of the RI measurement will be $<{7\cdot 10}^{-6}$.

## 4. Discussion and Conclusions

For the first time, a refractometer was considered as a device that made it possible to determine the difference between the refractive indices n_3_ and n_4_ of two liquids, where the refractive index of one of them (n_3_) was known [3]. However, later refractometers began to be classified as a differential type, meaning that they allow the measurement of small changes in the RI of liquid around a known (calibrated) value (n_r_). These devices are based, as a rule, on the standard measurement of the critical angle (or the angle of total internal reflection) θ_c_ = sin^−1^(n_1_/n_2_), when illuminating the interface between the measuring triangular prism and the liquid with a diverging beam of monochromatic light [2]. The angle θ_c_ specifies the position of the interface between the light and shadow in the transmitted (or reflected) beam of light on the recording element—a linear set of photodiodes (CCD sensor). Thus, the accuracy of measuring the refractive index is determined by the accuracy of measuring the position of this boundary. McClimans et al. [13] showed that the position of the boundary was determined not only by the number of the corresponding pixel (with a diameter of 25 µm) but also by the amplitude of its signal. It was assumed that the boundary of light and shadow is so clear that the amplitude of the pixel’s photo- response changes noticeably when the boundary moves along the pixel surface caused by a change in the refractive index. According to the authors, this approach makes it possible to measure the refractive index with an accuracy of no worse than $10^{-6}$. Some features of the use of differential critical angle refractometers in the case of absorbing media are considered in [14]. As point out by Zilio [15], the light from the laser diode passes through a polarizer and is then converted into a divergent beam using a semi-cylindrical lens, the flat surface of which is in contact with the liquid under study. The beam reflected from the flat surface of the lens passes through a polarizer–analyzer before hitting the sensor—a CCD web camera. When reflected from the flat surface of the lens at an angle θ_c_, the polarization of the light changes, as a result of which the analyzer does not transmit the reflected light, and the sensor registers a dip in signal intensity. The signal minimum corresponds to a specific pixel number. When the refractive index of the liquid under study changes, the value of θ_c_ and the corresponding pixel number change. This is the basis for calibrating the device using liquids with known refractive indices. The minimum measurable change in the refractive index using this method is ~$10^{-5}$. The considered methods, as far as we know, have not found wide application. It should be noted that industrially produced refractometers (see, for example, [15]) use measuring cells [3], the geometry of which is shown in Figure 1. The improvements relate mainly to the mathematical methods of signal processing [4,16]. Gong et al. [17] proposed to use a reflective mirror in the measuring cell [3], due to which the light rays passed through it twice. Hence, it was possible to improve the accuracy of the refractive index measurements to values ~$10^{-6}$. However, to achieve this result, they used a rather complex optical scheme, in our opinion.

In this research, we investigated the construction of a differential refractometer comprising two chambers, each with plane-parallel plates as its measuring components. We opted for rectangular cuvettes for their ease of production. The cuvettes’ geometry can be freely altered under a single condition: ensuring optical alignment between the laser emission and the measuring plate. Moreover, these cuvettes can be adapted for measuring fluids in motion. Utilizing the same laser pulse, sensor data from both the solution-filled and solvent-filled cuvettes were obtained, effectively nullifying any potential impact from laser radiation fluctuations. We gauged the refractive index measurement sensitivity based on the smallest discernible signal shift, equivalent to one sensor pixel’s displacement. Nevertheless, the preceding section delineates a methodology for pinpointing signal “coordinates”, enabling detection of the signal displacement at intervals finer than the pixel spacing on the sensor. This question, that is, the question of the real sensitivity of the proposed refractometer, requires additional study in future work.

The emergence of an advanced highly sensitive differential refractometer holds promise for precisely gauging variations in the refractive index across diverse settings and scenarios (e.g., indoor versus outdoor environments). Presently, humanity grapples with an escalating crisis of microplastic (MP) contamination in the environment [18], posing severe threats to human health and the broader ecosystem. MP pollutants have permeated all surveyed environmental mediums, infiltrating various trophic levels within the food web [19]. Within the realm of MPs, a nano-sized fraction known as nanoplastic (NPL) exists, ranging from 1 to 100 nanometers, potentially harboring unique nano-specific properties that amplify the biological risks [20]. Current ecotoxicological research underscores freshwater organisms as particularly vulnerable to the stressors posed by MP ingestion [21], though our overall understanding of MP’s biological impacts remains rudimentary [22]. Advancements in MP toxicity research face significant hurdles in the detection and quantification of both MPs and their nano-scale counterparts [23]. Their organic composition and minuscule size render distinguishing NPLs from naturally occurring particles a formidable task, necessitating the urgent development of tailored methodologies to bridge this analytical gap.

In conclusion, the high sensitivity (<7 · $10^{-6}$) and novel constructive solutions of the represented differential refractometer could serve as an objective background to propose it as a high-sensitivity sensing tool that can be used for mobile detection of nanoparticles in liquid samples, as well as to determine the level of environmental nano-pollution (e.g., in water, including rain and snow). Further studies should be aimed at refining the sensitivity assessments and exploring additional practical applications of this innovative refractometer design.

## Data Availability

The original contributions presented in the study are included in the article material, further inquiries can be directed to the corresponding author.
